# “I think of it as planting seeds”: challenging patient-provider discussions about COVID-19 vaccination: a qualitative study

**DOI:** 10.1186/s12875-025-03035-1

**Published:** 2025-10-28

**Authors:** S. Michelle Driedger, Ryan Maier, Colleen Metge, Alan Katz, Alex Singer

**Affiliations:** 1 College of Community and Global Health, Rady Faculty of Health Sciences , University of Manitoba, Winnipeg, MB R3E 0W2 Canada; 2 Max Rady College of Medicine, Family Medicine, Manitoba Centre for Health Policy, University of Manitoba, Winnipeg, MB R3E 0W2 Canada

**Keywords:** Infectious disease, Canada, Immunization, Pandemic, Decision-making

## Abstract

**Background:**

Vaccination has been essential in mitigating the worst effects of the COVID-19 pandemic in Canada. Healthcare providers can play a crucial role in promoting COVID-19 vaccination by discussing immunization, addressing patients’ questions, and providing them with relevant information. However, some segments of the public remained hesitant towards COVID-19 vaccination during the pandemic, reflecting an information environment crowded with misinformation and political polarization. This study examines challenging clinical discussions that healthcare providers had with patients hesitant about COVID-19 vaccines. It focuses on obstacles to fruitful conversations and strategies to overcome them, which can extend into ongoing vaccine-related conversations outside a pandemic context.

**Methods:**

Researchers conducted individual interviews with ten healthcare providers during the pandemic (January-May 2022) in the province of Manitoba, Canada. Participants were recruited using invitations distributed via professional organizations and networks. The recruited sample included primary care physicians, nurse practitioners, and a specialist provider who had recently discussed the COVID-19 vaccine with patients. Study participants were asked about their challenging conversations regarding the COVID-19 vaccine with patients and how they navigated these experiences. The resulting data were analyzed using NVivo12 to capture and organize salient themes.

**Results:**

Healthcare providers reported that COVID-19 vaccines have prompted new forms of vaccine hesitancy and resistance compared to existing vaccines, particularly due to concerns about the integrity of the vaccine (e.g., vaccine novelty, ingredients) or related public policy (i.e., vaccine mandates). Providers reported a significant rise in hostility from patients who were staunchly hesitant and experienced moral injury, burnout, and an emotional toll from witnessing disregard for public health. Participants indicated that they attempted to employ motivational interviewing strategies and shared decision-making and voiced desires for further training in such approaches. Some participants found mixed success with using decision aids or used improvised strategies to facilitate discussions.

**Conclusion:**

Motivational interviewing and shared decision-making strategies proved valuable to healthcare providers in navigating challenging discussions, addressing/acknowledging patient concerns, and preserving relationships. Healthcare providers need to be better supported with training in these strategies and in navigating the moral/emotional/physical consequences of experiencing a global health crisis in clinical settings.

**Supplementary Information:**

The online version contains supplementary material available at 10.1186/s12875-025-03035-1.

## Introduction

Vaccines have been a critical factor in the global effort to mitigate the worst effects of the COVID-19 pandemic. These effects include mortality, morbidity/severity, as well as the related social/economic disruptions resulting from overwhelmed health systems that require population-level restrictions to reduce viral transmission. Although proven to be safe and effective, COVID-19 vaccines prompted relatively high levels of hesitancy, misinformation (i.e., spreading misleading or unintentionally false information that runs counter to scientific consensus), dis-information (i.e., spreading intentionally false information) [1], political polarization, and significant public controversy over issues of perceived vaccine risk, lack of testing, mandates, and distrust of related entities of government, public health organizations, and pharmaceutical companies [2, 3]. Healthcare providers (HCPs, e.g., family physicians, nurse practitioners, and specialist doctors) tend to be trusted sources of health information, whose professional recommendations can increase the uptake of COVID-19 vaccines [4]. There is limited research that qualitatively explores the clinical dynamics of HCP-patient COVID-19 vaccine discussions from the provider’s perspective [5]. This study investigates the challenging conversations that HCPs in the Canadian province of Manitoba had with their patients about COVID-19 vaccines and documents different strategies used by HCPs to help increase the success of these conversations.

## Background

As COVID-19 vaccination campaigns rolled out across the globe, expert commentary promoted the role of primary HCPs as “vaccine ambassadors” [6, 7]. In most clinical settings, primary care HCPs are typically the first point of contact with health systems and often answer the public’s questions about vaccinations and respond to questions related to public health directives and recommendations.

In Canada, healthcare services (including primary healthcare) are generally provided to populations by provincial/territorial government authorities. However, the COVID-19 pandemic added unprecedented pressure on Canada’s already stressed and under-resourced health systems [8]. For example, primary care clinics had to significantly modify their practice activities (i.e., virtual care) to protect staff and patients and stay current with the latest pandemic/vaccine information and policies, all while continuing to provide necessary care to populations [9, 10]. The additional stress and strained resources contributed to soaring rates of reported burnout, isolation, and depression among primary HCPs around the world [9, 11, 12].

Vaccine hesitancy has been on the rise among segments of the public in recent decades [13], and HCPs have long been fielding concerns about vaccines such as debunked connections between Mumps, Measles, Rubella (MMR) vaccines and autism or distrust of medical/pharmaceutical industries [14]. During the pandemic, opposition/hesitancy to COVID-19 vaccines primarily coalesced around perceptions of disproportionate pandemic response measures, fears of vaccine safety (i.e., novel mRNA technology and unknown side effects), and concerns over the right to autonomous health decisions [15].

As the front line of health systems, (primary) HCPs found themselves on the receiving end of an increasing amount of the public’s ire, facing accusations of dishonesty, haphazardly promoting unproven vaccines, denying unproven alternative interventions, and helping to remove the public’s freedoms [16, 17] – with some doctors facing increasing personal harassment inside and outside clinics and hospitals [18]. Such accusations often came from more extreme right-wing, religious, and conservative political ideologies [19]. Religious and politically conservative views and low risk perceptions shaped public hesitancy in traditionally low-vaccinating areas, such as the southern regions of the Canadian province of Manitoba [20]. Along with an often overwhelming and rapidly evolving pandemic information environment, these factors increased the potential for challenging interactions for HCPs when discussing COVID-19 vaccination with hesitant patients.

Recommendations emerged promoting the use of ‘motivational interviewing’ as a promising strategy to address COVID-19 hesitancy and avoid confrontational or paternalistic interactions while building trust with patients [21–24]. Motivational interviewing probes patients’ core values/beliefs and promotes dialogue and collaboration in creating a ‘safe space’ for patients with compassion, empathy, non-judgment, cultural humility, and sincerity [25, 26]. Motivational interviewing respects patient autonomy and intends to meet patients at their readiness to discuss potentially contentious topics such as vaccination – acknowledging that each engagement may form one step in a multi-visit conversation [27]. While motivational interviewing is a promising patient-centered approach intended to build trust through empathy, its effectiveness in discussions regarding COVID-19 vaccination (or vaccination more broadly) has yet to be systematically demonstrated [28].

While conceptually distinct from motivational interviewing, ‘shared decision-making’ similarly promotes respect for patient autonomy, leveraging multi-visit discussions, collaborative problem-solving, and probing of patient values – with added emphasis on sharing the risks and benefits of the available evidence and use of decision aids to promote understanding and comprehension [29, 30]. Shared decision-making has also been encouraged to facilitate discussions regarding COVID-19 vaccination [31], with some advocating for a combination of motivational interviewing and shared decision-making [30]. In either case, the strategies focus less on the actual outcome of the discussion and more on whether a conversation takes place at all [32]. Additional practice recommendations included procedural strategies, such as pre-appointment vaccine status checks [33], and further training in discussion approaches, including motivational interviewing and shared decision-making [6].

To date, relatively limited research has qualitatively examined the perspectives of HCPs in their clinical discussions of COVID-19 vaccines, especially within Canada. For example, one Canadian study of a small set of HCP interviews found that HCPs may not be sufficiently engaging their patients in COVID-19 vaccine discussions or were avoiding the topic [5]. Several U.S.-based studies reported that HCPs expressed frustration with addressing misinformation among vaccine-hesitant patients yet tried to promote vaccination using trust-building strategies, such as motivational interviewing, expressing empathy, communicating over multiple visits, or using information aids if available [34–36]. Our study builds on these studies to further explore HCPs’ experiences of challenging COVID-19 vaccine discussions with patients. This topic remains relevant as the COVID-19 virus continues to circulate globally, and the ongoing utility of vaccination as a lifesaving measure will continue for other diseases.

## Methods

This study is part of a broader investigation examining challenging discussions between HCPs and patients about routine issues, such as pain. When the pandemic arrived and routine HCP patient visits for common issues declined, the study researchers adapted the project aims and shifted their focus to examining difficult conversations about COVID-19 vaccines. Distribution of COVID-19 vaccines began in December 2020, and by late September 2021, 79.4% of Manitobans were fully vaccinated [37]. Public health then focused on promoting uptake among those who remained unvaccinated and were more hesitant or resistant to vaccination. To further increase uptake, in September 2021 (the same time vaccine mandates went into effect in the province) [38], the Manitoba government invested in a $14 million initiative to support HCPs in identifying, contacting, and having conversations with the unvaccinated within their practice rosters [39]. With HCPs in the province engaging the hesitant and unvaccinated, researchers aimed to recruit HCPs and explore the challenging factors they were having in these conversations about COVID-19 vaccines. Researchers received ethics approval for the adapted protocols from the University of Manitoba Health Research Ethics Board in December 2021 (reference number: H2019:040).

HCPs have long been a challenging population to recruit, often due to limited time availability amid busy schedules [40–42]. Researchers pursued a broad and open recruitment strategy to offset any recruitment challenges that may be further exacerbated by the pandemic and capitalize on the limited time of the provincial initiative promoting engagement with unvaccinated patients. To be included, HCPs need to have engaged in discussions with patients regarding COVID-19 vaccination. We also did not screen participants to achieve a preset balance between provider types of HCPs (e.g., family physicians; nurse practitioners) because we did not aim to compare experiences between provider types. Recruitment involved study invitations signed by two HCP team members (AS and AK), distributed via networks (e.g., Manitoba Primary Care Research Network) and newsletters of HCP professional organizations in Manitoba (e.g., Doctors Manitoba).

Study invitations yielded only 10 HCPs who responded to participate and thus data collection consisted of 10 individual interviews with HCPs between January and May of 2022 (approximately between the fourth and fifth COVID-19 wave in Manitoba [43]; provincial COVID-19 mandates ended on March 1, 2022, with eight interviews before the end of mandates and two afterward). As such, the study timeframe roughly followed from after the start of the provincial initiative in September 2021 to just past the end of provincial vaccine mandates in March 2022. Data saturation can vary in the number of participants involved in a study and can depend on the contextual constraints and the purpose of the study [44]. The benefit of qualitative interviews is the provision of depth, where breadth may be limited. The data collected – while not saturated to the point of having ‘heard it all’ from a limited participant cohort – was sufficient [44] within the contextual constraints (i.e., at a time when HCPs were committing extra time towards patient engagement and vaccine promotion) to provide a description of salient themes and challenges faced by HCPs in their COVID-19 vaccine-related conversations with patients.

Interviews began by obtaining informed consent and lasted approximately 30 minutes. Interviews took place via video/teleconference and were facilitated in English by the study lead (SMD). SMD is a Canadian researcher with over two decades of experience as a qualitative interviewer, including experience interviewing HCPs, focusing on trust and risk communication. Interviews included topics of provider experiences with pre-COVID-19 vaccination discussions with patients (e.g., childhood/seasonal vaccines), COVID-19 vaccine discussions, facilitators/obstacles to fruitful discussions, personal and practice challenges during the pandemic, and strategies used by participants to engage in these discussions (see appended Interview Guide).

Following each interview, SMD compiled detailed notes of impressions and initial analysis of HCP perspectives. These notes formed the early analysis phase and indicated points for exploration in subsequent interviews. Completed interviews were transcribed, audio-verified, and entered into NVivo12 qualitative software for coding and analysis. RM and SMD initially reviewed a sample of transcripts (i.e., early, middle, and later interviews, to capture progression of thematic ideas in earlier interviews and early analysis through exploration with subsequent interviews over time, plus to capture views of participants with different professional specializations) and identified salient themes through initial iterative engagement with surface content [45] (e.g., dis/trust, strategies, challenges, use of decision aids, burnout/exhaustion, routine/COVID-19 vaccines, patient concerns, hesitancy, hostility). Emerging themes were then discussed with the broader research team (including clinician-researchers) to challenge any underlying assumptions and confirm that themes reflected the realities of clinical practice – these measures helped to ensure rigour and interpretive consistency. The themes then comprised the coding framework with which RM completed the coding of the dataset – engaging with SMD and the study team to resolve coding and analysis decisions through discussion. Researchers also developed a brief report summarizing the core themes emerging from the data and sent the report to HCP participants with the opportunity to challenge interpretations and provide feedback. For this study, we present a descriptive analysis of the core themes arising from data.

## Findings

Participant interviewees included seven family physicians, two nurse practitioners, and a specialist doctor (oncologist). Five family physicians, one nurse practitioner, and one specialist worked in the urban capital city of Winnipeg. One nurse practitioner and two family physicians worked in non-urban/rural regions of Manitoba. Three physicians and one nurse practitioner were women; the six remaining participants were men. Here, we present three inter-related themes that emerged from the data, which focus on changing patient perspectives, discussion strategies, and repercussions on HCPs. First, we look at how patient perspectives and manifestations of vaccine hesitancy changed between pre-pandemic times for routine (e.g., childhood) vaccines and then for COVID-19 vaccines during the pandemic. Next, we examine communication strategies used by HCPs to potentially influence patient perspectives, including discursive methods such as motivational interviewing and decision aids. Finally, we highlight the harmful physical and emotional repercussions of challenging conversations with patients about COVID-19 vaccines on HCPs. Representative quotes reflective of themes are found in respective subsections. While we include the practice type (i.e., family physician) and geographic context (urban/rural), names of HCPs and identifying information have been removed to preserve anonymity, given the relatively small population of HCPs practicing in Manitoba and in smaller regions of the province.

### This vaccine was different: patient perspectives on routine vs. COVID-19 vaccines

Participants recounted that before the pandemic, most people generally accepted routinely available vaccines, yet a small subset exists among them for whom hesitancy or resistance is more pronounced. Aside from the small number of patients described by HCPs as staunchly anti-vaccine, HCPs noted that most people expressing hesitancy are receptive to at least a discussion about immunizations. HCPs who practiced in traditionally low-vaccinating areas of Manitoba reported relatively higher proportions of patients who avoided vaccines and discussions of them. HCPs noted that opposition to standard vaccines is usually rooted in fear of side effects and ingredients, religious beliefs, and distrust of pharmaceutical companies, government, and health systems. Generally, HCPs reported that conversations about routine vaccinations were usually amicable, predictable, and manageable.

I find up until COVID-19 most people were quite happy to get vaccinations […] But you know, by the time you’ve had your fourth shot of the same thing people don’t – they’re most interested in it on their first child, the first time they’ve seen it. But if they’ve had four kids and you’re like, ‘we’re giving him an MMR,’ they’re like, ‘yeah, yeah.’ - Family Physician 1, Urban.

[In the regional health authority] where I practice, like, the vaccination rates are close to the lowest in the country. Either there’s no conversation at all and they just say, ‘of course I’m going to get my kid vaccinated,’ or you explain everything to them and they still don’t want to. They’re worried that there’s autism or they’re worried that there’s mercury. They have this general distrust. I think our childhood vaccination rate was, like, 60%, and the COVID vaccination rate is around 50%, so I think that’s fairly similar populations. – Family Physician 5, Rural.

However, HCPs reported that the pandemic and consequent conversations about COVID-19 vaccines completely upended the usually predictable course of routine vaccination discussions. As with familiar vaccines, many patients were willing to accept COVID-19 vaccines. Yet, the subset of hesitant or strongly opposed people they observed appeared more extensive and more vocal than before the pandemic, and they displayed characteristics that reflect traditional sources of hesitancy (e.g., distrust) as well as those unique to COVID-19 vaccines (e.g., fear of mRNA technology; hostility to mandates).

I was facing a surprising amount of backlash from people who you wouldn’t traditionally consider anti-vaxxers who would take up regular vaccines […] They say, ‘I’m not an anti-vaxxer, but…’ – and I’m like, hmm […] There has emerged a subset of people who are simply opposed to vaccines because they’re mandated. These are people who don’t like being told what to do, and that crystalizes that hesitancy into an absolute flat-out refusal. And those are people I really can’t have a conversation with because their views are no longer based on fear or questions or experiences, they’re opposed philosophically simply because someone says to do it. – Family Physician 3, Urban.

A lot are not in that contemplative or pre-contemplative [mindset]. They’ve made their mind up. It would be very hard to get them to re-examine their belief. I find a lot of them are trying to get me to re-examine my beliefs […] And then they start, ‘so would you give me ivermectin?’ I don’t think I’ve been able to change anyone’s mind for a while. – Family Physician 1, Urban.

Some HCPs described seeing patients who were explicitly hostile to COVID-19 vaccination and unwilling to discuss the matter – even among patients who generally accepted other vaccines. HCPs’ descriptions of the most resistant patients reflected a rough typology of oppositional characteristics. HCPs identified some patients reacting to any paternalist authority ‘telling them what to do’ (via recommendations or mandates). HCPs noted that other strongly anti-vaccination patients expressed more attitudinal distrust of health systems, doctors, scientists, and political authorities and sometimes referenced conspiracy theories and false information. HCPs identified certain brands of political conservativism (e.g., libertarianism, anti-government, partisanship) or the confusing media environment as drivers of opposition and hesitancy. Some HCPs described a dissonance among patients who would refuse the COVID-19 vaccine because they distrusted the science and medicine behind it. However, they would readily accept other pharmaceutical interventions with less robust testing regimes or higher risk profiles (e.g., ivermectin). Some HCPs working in historically under-vaccinated areas of Manitoba also witnessed hesitancy in their practices among patients who perceived that there was low pandemic risk in rural areas, cited religious influences, and distrusted pandemic messaging from those considered ‘outsiders’ (i.e., pandemic managers in the provincial capital). Many agreed that hesitancy often became connected to issues not always integral to the vaccines (e.g., religion and partisanship) and that talking to patients who had their minds made up and would not accept a discussion was highly challenging.

I would say there’s two kind of flavors to them [vaccine resisters]. One would be some people are just outright hostile. If you bring it up they already have their guard up: ‘well that’s my personal choice,’ or, ‘I don’t want to talk about that.’ There are some people who will listen politely and say, ‘still not interested.’ Then there’s a very small number who seem to be taking in information and recognizing that they’ve been misinformed. […] Sometimes the conversations devolve in bizarre political tangents [or identifying concerns] that are correctable. Like they’ll say they don’t know what’s in it. So you go to the CDC and here’s the ingredients. [But they aren’t interested in correctable information] and they move on to the next talking point. – Family Physician 5, Rural.

There’s a different feel between rural and urban […]. Our vaccination rates are really low. So it’s not as simple as just having a conversation. So the hesitant folks, leaning more to anti-vax kind of folks, they’ll come with the same kind of ‘I read this on Facebook,’ or ‘I had this conversation,’ right? So wrapped in that is also the ‘I don’t want to wear a mask. I don’t want all this. I’d hate the impact on my employment.’ So it becomes bigger than just vaccines. – Nurse Practitioner 2, Rural.

With COVID vaccinations, the difficult ones are irrational. I’ve had patients and it’s like, ‘well, I notice you’ve had vaccines before. What’s different about this one?’ They have reasons and arguments, but try to explain to them how that’s not based in facts and they get very irrational and angry. The fervent ones, you’re almost dealing with a cult or religious movement. They see physicians, government, and the media as a group of bad guys. I’ll talk about the vaccine then and I’ll ask them, ‘why do you come here? You don’t believe what I’m saying, why do you want my opinion?’ ‘Well I wanted a prescription for a chest cold.’ You don’t know anything about that drug either but you’re OK with me prescribing that drug. – Family Physician 6, Urban.

### HCP strategies for negotiating challenging vaccination discussions

Most participants shared patient-focused strategies – while not necessarily using the exact formal terms – that fit within frameworks of motivational interviewing and shared decision-making. Some participants began incorporating a new workflow habit of checking their patients’ vaccination status before or during appointments to cue whether or not to address the topic. Broaching the topic allowed HCPs to gauge the potential resistance level and whether a fulsome discussion was appropriate or possible.

When I prepare for my patients every day I go through […their] eChart’s immunization history – [to] make sure they’ve had their COVID shots lately. I check their tetanus and pneumonia, and I try to bring up the shingles shot if they haven’t done that. It takes probably an extra half hour a day to prepare. – Family Physician 1, Urban.

Participants commonly reported attempting trust-based approaches as they entered vaccine conversations with hesitant patients: being open, compassionate, non-judgmental, respectful, and sincere in their curiosity while soliciting a patient’s fears, concerns, and motivations. They would then speak towards and leverage those issues (e.g., allay fears of side effects, reframe motivations) and provide vaccine information while ultimately recommending vaccination. Many also agreed that allowing patients to share their concerns in a ‘safe space’ helps anxious patients feel heard and validated. Such collegial space is an end in its own right – as it builds the trust necessary for other crucial aspects of their care – but it is important for trauma-informed care, particularly when working with patients who face barriers to access.

It’s really important to be clear that you’re coming from a position of caring about them and their wellbeing. So I always make sure that I express that explicitly, ‘this isn’t me trying to follow instructions by the government or check off a box on your chart, this is about me being worried about your diabetes and your age and how COVID might affect you.’ We try to always mention that ‘regardless of your decision you are welcome here and this is not going to affect our relationship in any way. We’re here just to make sure you have the proper information and we want to understand your decision. So what are your values, what’s important to you, what are you fearful of?’ And then gauging the readiness, this is an important trauma-informed technique. Seeing where they’re at that day in their openness to having a conversation. Prefacing by saying, ‘would it be OK if we had a discussion about this for a few minutes?’ It’s not uncommon for folks to say, ‘no, I don’t want to talk about that today.’ And that’s great to know because if you just launched into that conversation, you may be actually turning people more against that conversation. So if they say ‘no, I don’t want the vaccine, I don’t believe in it,’ then saying, ‘well, help me understand how you got to that conclusion. This isn’t about me trying to convince you, I just want to just understand because it helps me know how to support you with many other healthcare decisions that might come up in our journey.’ – Family Physician 2, Urban.

Many participants described a longitudinal approach to vaccine discussions, where each opportunity for discussion was one step in a ‘long game,’ and each conversation was another chance to advance the dialogue or continue to gauge patients’ readiness – a process one HCP described as “planting seeds.”

Part of my conversation with folks who are pretty firm in not getting the vaccine is I’ll say, ‘I will probably ask you this again or touch base with you intermittently just to see if you have any change of heart.’ I think of it as planting seeds. A lot of what I do in primary care is I plant seeds. I plant seeds about change, I plant seeds about smoking cessation, I plant seeds – right? And sometimes my job is longitudinally to nurture and water those seeds. So, in the patients who have conversations with me, whether or not they sought out the vaccine, many of them were coming back with second- and third-round questions. And so for those individuals it wasn’t usually a single call or single conversation intervention where they would get to a place where they had crystalizing intent. – Family Physician 3, Urban.

HCPs wanted to remain mindful that they treat people who are more than just their opinions on vaccines. HCPs reported that this approach sometimes successfully facilitated a patient’s vaccination decision. Other times, they noted less success with the most resistant but that it preserved collegiality and left room for revisiting the topic in the future.

With patients presenting with highly anti-vaccine opinions, some HCPs practiced what one called ‘informed refusal,’ where they respected the patients’ right to oppose vaccination but felt obliged to ensure that the patients listened to them share some information about COVID-19 vaccines. HCPs often recorded in patient charts whether such conversations took place (for revisiting next time if necessary or appropriate). This procedure became a default approach for HCPs who felt pushing a conversation would not be fruitful but wanted to acknowledge the subject and pass on pertinent information. Other notable strategies included integrating vaccination conversations as part of routine patient care, providing examples of different kinds of safety-related mandates (e.g., seatbelts), and using humor to keep the tone of discussions light. Other HCPs recommended that COVID-19 vaccine discussions with patients would have been helped – or could be helped in the future – with further training on handling challenging vaccination discussions, including training in Motivational Interviewing.

My job is to have an informed conversation, help them understand the medical perspective, ask what people’s concerns are, and if they choose not to take me up on the offer to vaccinate, I need them to at least acknowledge the fact. I do something that I consider like an informed refusal. It’s not enough that you just say ‘no thanks,’ like you’re passing on *hors d’oeuvres* at a party. That means sometimes you just sit there and you have to let me say my thing, and then say, ‘OK, thanks for telling me, and still no thank you.’ And provided that they have allowed me to share the information, from there I respect whatever choices they make. My job is to be their expert advisor. If I can maintain a safe space where they feel they can ask a question without being judged then they’re more likely to feel safe to ask questions of me rather than get their information from questionable sources. – Family Physician 3, Urban.

There was a motivational interviewing session offered, I think from a psychologist in Toronto, which was very helpful to learn, specifically regarding COVID. But I need training, practice, and feedback. I don’t feel very confident. I have used motivational interviewing a lot with smoking cessation, so that I feel like I can implement it, but I can’t do it with a lot of other things. – Nurse Practitioner 1, Urban.

Some participants described using various tools, like information aids or media, to connect patients with information about COVID-19 vaccines and facilitate decision-making. Some HCPs attempted to point patients toward more credible websites, and one reported collaboratively ‘Googling’ information during appointments and discussing what they found. Several HCPs used available COVID-19 vaccine information sheets downloadable from government health sites, which they found valuable as they were locally sourced and frequently updated as vaccine information and policies were evolving. One participant was more critical of how the provincial government website shared COVID-19 data, indicating that other provinces produced less confusing data for public consumption. Another HCP reported wishing they had COVID-19 informational handouts available for patients.

They’ve got these great information sheets, Shared Health and Manitoba Health. And at the beginning where people want to know, is it safe for pregnant women, and they had these frequent updates which was really nice because it was showing Manitoba data. – Family Physician 1, Urban.

I will direct them to what I consider reasonably trustworthy websites, Mayo Clinic and stuff. I do tell them just make sure that it’s a bona fide, respectable organization. Don’t go to Billy Bob’s website to get information. That’s one of the things that I wish we had better – we had decent handouts with, you know, updated stats on – they change every week and you can hand those things to them and say look who’s vaccinated and not. – Family Physician 6, Urban.

Some resources are more friendly than others. I think that Manitoba has presented their numbers very poorly. Whereas if you look at what Alberta has done, they have this very easy to read - age, triple vaccinated, double vaccinated, unvaccinated, and rates per 100,000. So then it’s very easy for someone to see how well things are working. – Family Physician 5, Rural.

Some participants noted that dealing with oppositional patients was, at times, frustrating and admitted that their tolerance for dealing with conspiracy theories and false information challenged their optimal levels of engagement with some individuals.

I was just suturing a laceration in the ER, asked him about vaccination, ‘no I’m not vaccinated.’ So we went down a few things, and he didn’t believe that humans had ever been to space and these were just things that the elites told you to keep you in line. So at that point you give up on thinking that you’re going to make any headway. But, I think that as long as it seems to be still on kind of a cordial level and they’re not saying anything that’s, you know, insane, then I kind of carry on with a bit of back and forth. – Family Physician 5, Rural.

HCPs reflected that providing informational handouts or direction was often an acceptable middle ground in preserving amicable relationships with oppositional patients while providing accurate information to attempt to balance some of the false information circulating. One participant acknowledged that patients often like to leave their clinic with ‘something’ in hand, and they had developed their own ‘sticky notes’ or ‘take-away cards’ to support clinical conversations that patients could take with them from a visit. This method was a way for them to provide passive information about clinical topics, health promotion tips, and other issues that do not require a prescription but in a way that was still empowering and left the door open to future discussions.

The issues that would come back repeatedly when I was early in practice was that so many conditions did not require a prescription. It was common-sense care. If you have a cold you should drink warm drinks, rest, use lozenges or saltwater gargles. But people don’t like to leave empty handed. So I would write stuff down on sticky notes. And then I realized I was writing the same stuff every time. So went [to a local printer], and I printed a whole series called Dr. [Participant]’s ‘What to Do Series.’ And they’re all evidence-based conservative care stuff. What to do when your kid has a virus. Things not to ignore, red flags, when to talk to me, right? When to come back. Like, they’re so used to going to the doctor, get a prescription, it’s like, no. I have a whole sticky note of crisis resources because it’s just convenient to give to people. – Family Physician 3, Urban.

### Moral injury from COVID-19: going beyond physician wellbeing and ‘burnout’

All HCPs we interviewed described the high toll that the pandemic had on their practice. While many described an under-resourced health system even before the pandemic and pointed to the stress of changes to practice procedures, they all directed attention on the dramatic increases in hostility directed at them and their profession by patients and the public. Most reported losing job satisfaction and experiencing burnout from talking to more challenging patients, negotiating a noisy (mis)information environment, and having patients selectively choose what advice they were willing to believe. One participant characterized the indifference of some resistant patients as an experience of “moral injury” (i.e., being a witness to behaviors that go against one’s sense of moral responsibility) since vaccine refusal has potentially harmful consequences, leaving them emotionally drained. Some care providers shared steps that they are taking to protect themselves and their physical/mental wellbeing, such as engaging in meditative breathing exercises. Some HCPs feared the damage that the pandemic had wrought on HCPs and the possible consequences for physician retention in some places (especially historically under-vaccinated areas).

It makes it much less enjoyable because it’s hard not to take it as a personal affront when someone with a grade 8 education thinks they know more about vaccines than you. Why are you coming to my office? If you know so much go home and treat yourself. It’s also a frustration to see that these people are causing so much harm that there is no social accountability for that at this point, or very little. Prior to Omicron almost all of the hospitalizations, almost all of the ICU stays were unvaccinated, and they’re still a disproportionate number to the percent of the population, and we were letting this 10% of society collapse our health care system. – Family Physician, Rural.

It’s very difficult because we get all sorts of nasty behavior. And all we’re doing is asking them some questions on why you’re not vaccinated. There are the other ones who will get very angry, like, ‘it’s none of your business, how dare you ask me about that?’ Well, it’s my job, perhaps I’m supposed to ask you about that stuff and provide the information and answer questions. Some of them get furious. We had one lady accusing us being like Nazi war criminals. Forcing people to get vaccinated. I’m not forcing you to do anything, I’m just recommending you that you should, and the reasons why. It’s hard, but we try. – Family Physician 6, Urban.

We’re really seeing terrible stuff and we’re undergoing terrible burdens ourselves and it’s just such an incredible moral injury to have that kind of resistance to what should be a community benefit and our jobs as citizens’ responsibility. So, I realized that there was no way I could have a conversation that would open doors with people when that was my emotional response that I would go to. I made some proactive changes before people even knew stuff was happening. So I had actually back probably end of March 2020, very shortly after our first case, I said you know what? This is what’s coming. I’m going to put in some boundaries in my practice in place to protect myself because I know we’re looking at a years-long event, and I know that I’m probably going to burn out. – Family Physician 3, Urban.

We had so many problems with people refusing to wear masks. Anything they can do to gum up the works, they do. They refuse COVID swabs at admission […] And it’s behavior that you couldn’t tolerate from everyone. I think there is some evidence now that this is having an effect in [Regional Health Authority] on recruitment, like, we actually have hard numbers showing that we’re retaining a lower percentage of our residents now versus pre-pandemic. And so I think that people are underestimating the effect on morale. – Family Physician 5, Rural.

## Discussion

According to participants, while they had grappled with prevailing trends of vaccine hesitancy before, with COVID-19 vaccines, the typical patterns changed to include a portion of hesitant or oppositional patients who were much more hostile, distrustful of doctors, and rigid in their views or resistant to information or discussion. This trend matches a unique COVID-specific hesitancy among the public compared to general vaccines described elsewhere [46, 47]. Participants found that COVID-19 vaccine hesitancy took on specific contextually-driven characteristics, including concerns over vaccine integrity (i.e., fear of unknown side effects, testing and approval, mRNA technology) along with new fringe conspiracy theories, partisan divides, opposition to mandates, and misleading COVID-19-specific information. HCPs practicing in historically under-vaccinated areas of the province (i.e., southern, rural regions of Manitoba) anticipated and witnessed similar developments, augmented by the religious beliefs of specific regions, low risk perceptions, and urban-rural divides. Other research has also found that fear of COVID-19 vaccine side effects, misinformation, misperceptions of risk, partisanship, religious influence, and conspiracy theories all played roles in driving hesitancy among populations [15, 19, 48, 49]. This study connects those perspectives to their articulations in clinical contexts and how they challenged subsequent COVID-19 vaccine discussions between HCPs and patients.

Experts correctly anticipated that HCPs would become de facto ‘vaccine ambassadors’ on the front line of the fight against the COVID-19 pandemic [6]. Study participants embraced this role and employed several strategies to facilitate collegial vaccine discussions with patients – in contrast to findings in a similar Canadian study [5]. Our participants described using strategies that fit within established frameworks of motivational interviewing (i.e., probing for patient values and concerns, being empathetic, non-judgmental) [25, 26] and shared decision-making (i.e., sharing benefits and risks, respecting patient values and autonomy) [29, 30] and anecdotally reported relative success in creating non-confrontational space for discussions, whether it led to achieving vaccine uptake or maintaining ‘open doors’ with the most resistant patients. In keeping with literature elsewhere [50, 51], HCPs recognized that – whether in the vein of motivational interviewing or shared decision-making – the crucial factor may not always be the ultimate decision made by the patient at that moment but is the process of sharing information and keeping the lines of communication open. Both motivational interviewing and shared decision-making have been promoted among clinical experts as potentially effective facilitators of COVID-19 discussions [23, 31], along with blending the two approaches [30]. Our participants’ positive reflections and desire for training in using these strategies support such recommendations.

Meeting patients at their level of understanding is a core aspect of trust-building strategies like motivational interviewing [52], and participating HCPs consistently attempted to maintain a disposition of non-judgmental compassion and empathy with hesitant patients. Rather than risk scuttling a relationship over differing perspectives, some HCPs endeavored to engage the patient in a conversational ‘long game.’ If a patient was particularly hesitant or did not want to talk further, HCPs anticipated re-checking vaccination status and revisiting the topic on subsequent visits, thus ensuring an ongoing safe environment for a patient to express concerns and for conversations to evolve. HCP anecdotes in this vein supports similar clinical recommendations that COVID-19 vaccine discussions can be more successful if they occur over time, as it takes time for people to reset expectations and attitudes [27, 53, 54]. This longitudinal approach to COVID-19 vaccine hesitancy also fits well with a ‘wait-and-see’ approach to COVID-19 vaccines that many hesitant people espoused [23, 47].

Even when faced with challenging or oppositional patients, HCPs still attempted to convey relevant information to ensure that it at least met the patient’s ears – a process one participant called ‘informed refusal.’ As an existing concept, ‘informed refusal’ relates primarily to childhood vaccines (often required for school attendance). It can be a formalized clinical mechanism for documenting that a patient has received information on risks/benefits when they refuse a mandated vaccine [55]. While we did not find similar recommendations in COVID-19 literature, some participants seemed to have implemented an ad hoc informal mechanism akin to ‘informed refusal.’ This approach recognizes the right of the patient to refuse a vaccine while ensuring a minimum of relevant information has been exchanged (similar to shared decision-making), and ‘informed refusal’ for clinical counseling with vaccine refusers has been promoted in the literature [56]. While a concept like ‘informed refusal’ does not seem to appear in related literature on motivational interviewing or shared decision-making for vaccine-hesitant patients [50], its recognition of patient autonomy reflects respect for patient choice while ensuring patients have the information to make decisions.

Shared decision-making often involves using information or decision aids [51]. Study participants only infrequently noted their use of aids to facilitate discussions or patient understanding. Several participants indicated a lack of success finding locally relevant, suitable materials and wished they had such materials to assist in vaccine discussions. Nevertheless, HCPs attempted to steer patients towards more credible internet sources and share some government data sources – although the quality of government materials seemed inconsistent across Canadian provinces. Using decision aids as part of shared decision-making can positively influence vaccine uptake [51]. Thus, the lack of (or desire for) such aids suggests that such materials could be potentially effective, particularly as COVID-19 becomes endemic. One participant’s use of self-developed ‘sticky notes’ for patients to take basic advice with them (conceptually similar to prescriptions for exercise [57]) provided a unique example of non-confrontational information distribution. As the pandemic moves into endemic phases and vaccines are no longer mandated, such non-confrontational information-sharing strategies may be helpful in future vaccination campaigns.

While similar qualitative research on HCP perspectives has been relatively rare to date, one study of U.K. general practitioners found that they faced increased COVID-19 vaccine concerns from patients, experienced frustration with the confusing information environment, and felt themselves the target of negative public perceptions and ‘GP bashing’ [58]. Our study participants echoed the U.K. practitioners’ experiences and reported being accused of deceit and corruption by patients, even in the clinical setting. Our participants also pointed to the sense of moral injury they felt when priorities of patient-centered care (respecting anti-vaccine choices) and protecting the broader public health (promoting vaccination) came into personal conflict [59]. Similar research has highlighted moral injury/distress among HCPs but focused more on difficult decisions about scarce resource allocation, unsafe work environments, and end-of-life patient care [60]. This study offers evidence highlighting the morally distressing dynamics between HCPs and the patients who resist the responsibility to protect the public through vaccination. This study further shows that – especially in under-vaccinated regions – experiences of moral injury may hamper the retention of HCPs who leave or avoid practicing in regions due to high levels of patient hostility. Thus, especially in a public health crisis, while motivational interviewing and shared decision-making may offer valuable practical techniques for facilitating difficult vaccine decisions, such methods may still leave unaddressed the moral repercussions HCPs can face when many patients exercise autonomous decisions that ultimately hold broad social implications.

Our findings provide qualitative support for physician surveys that commonly reflect a decline in wellbeing and increased reporting of burnout, isolation, depression, and exhaustion from increased workloads, practice adaptations, and facing public/patient ire [9, 11]. While existing literature tends to promote individualized wellness actions (e.g., yoga, meditation, self-help strategies) to help HCPs deal with times of pandemic stress [12], our participants also provided related practical examples of attempting to improve personal/practice wellbeing. These included added preparation time, breathing exercises, and using digital reminder mechanisms in medical records to prompt discussions.

This study has limitations. First, our small sample size of participants in the province of Manitoba limits the scope of generalizability, not only for the themes identified above but also for experiences of different types of HCPs involved in those discussions (e.g. family physicians, nurse practitioners, pharmacists) and HCPs practicing in other provincial or national jurisdictions – as different provinces implemented policies respective to their circumstances/priorities. As was anticipated, with many HCPs taking on increased workloads, plus added pandemic stress and limited time within the study’s timeframe, recruiting a large sample of HCPs was challenging. Second, this sample of participants likely reflected a degree of volunteer bias as participants likely had a particular interest in the research topic. Future research should attempt to capture a broader sample of interview participants and investigate later-stage pandemic developments. Lastly, while the data provided here has been exploratory, future research should systematically examine the efficacy of motivational interviewing and shared decision-making as strategies for facilitating pandemic-context vaccination discussions.

## Conclusion

Notwithstanding their many challenges, all HCPs reported approaching challenging discussions with the degrees of empathy, humility, and respect recommended for engaging with vaccine-hesitant patients during the COVID-19 pandemic. Their use of motivational interviewing and shared decision making – with attendant approaches of using ‘informed refusal’ or playing the ‘long game’ – helped them to broach and carry on with vaccine discussions that many of their patients did not want to have, illustrating the potential of these strategies in facilitating discussions. As the need for COVID-19 vaccine booster doses remains a critical public health recommendation (and, further along, as COVID-19 vaccines is folded into messaging alongside seasonal influenza campaigns), the strategies used by participants in this study may be helpful for other HCPs in their clinical environments. Further, their desire for additional training in approaches like motivational interviewing or improved decision/information aids signal valuable areas where support for HCPs is needed in the future. Additionally, future research in this vein, along with practicing HCPs and interested stakeholders, should consult health communication literature for further opportunities for training to support similar interventions and strategies.

## Supplementary Information

Supplementary Material 1.

## Data Availability

The raw data cannot be published online due to restrictions of confidentiality. Due to stipulations on confidentiality consented to by participants, de-identified datasets are also not available. Please note: all information, including the audio recordings, will be destroyed after ten years.
