# Supplementary material for: “I think of it as planting seeds”: challenging patient-provider discussions about COVID-19 vaccination: a qualitative study
Source: BMC Prim Care. 2025 Oct 28;26:326. doi: 10.1186/s12875-025-03035-1 (PMC12560340; doi:10.1186/s12875-025-03035-1)
Supplement: Supplementary file 1 — Supplementary Material 1. [file 12875_2025_3035_MOESM1_ESM.docx]

**Exploring patient-provider dialogue – fostering trust through joint clinical decision making for sub-study – “Challenging patient-provider discussions about COVID-19 vaccination” - Interview Guide**

**Funder:** Canadian Institutes of Health Research

**Study Site:** Winnipeg, Manitoba, Canada

**Institution:** University of Manitoba

[NOTE: Not all questions are asked exactly as they are written. As delivered by an experienced interviewer, interviews are very much like a conversation. Interviewees often bring up topics on their own, and probes are not automatically asked, but are there in case issues may not be brought up by interviewees to the primary questions]

## General (childhood, flu, etc.) vaccine questions

1. In your practice, do you typically have conversations with patients about vaccinations generally?
   1. [If participant says yes] What has been your general approach to managing a patient who is hesitant or refuses a vaccination when you recommend that they should have it?

*Probe/listen for (not always asked, if not brought up by interviewee in response to above questions)*:

- Does this happen often? Is a general approach possible? Or do you tailor your approach to each patient? For example, how do you approach patients who are ‘hesitant’ or those who are appear to be anti-vaccination?

## Covid-19 questions

1. What has been your approach to having a conversation with a patient who is hesitant or refuses a COVID-19 vaccine?

NOTE: spend time exploring how similar or different COVID-19 discussions might be from general vaccinations, depending on what participants have described in their own words.

*Probe (if not raised by participants)*:

- Do you have a general or tailored approach? For example, how have you approached patients who are ‘hesitant’ versus those who appear opposed to COVID vaccination?

**3.** [*This Question can follow each question above respectively, or follow both here*]

What kind of reactions from patients have you had when you had such conversations (for general or COVID-19 vaccines)?

*Probe/listen for – and explore only as relevant if not raised by the participants themselves from the open-ended question*:

- Were patients receptive to your recommendation? How did it differ (general vs COVID vaccines)? What impressions did you have from the encounter?
- When providing clinical recommendations, patients may bring into the discussion external influences (e.g. things patients read on the Internet, or things they heard from family/friends/politicians/other perceived ‘experts’, celebrity doctors, like Dr. Oz, etc). Were you able to communicate the clinical recommendation without having it challenged by ‘outside’ views?
- How have/would you handle(d) a patient who disagrees with your recommendations? What do you think has worked well and what has not?
- [*For COVID vaccination*] Has the different ‘kinds’ of COVID-19 vaccines available (Pzifer, Moderna, J&J, AZ) influenced your conversations in any way (e.g. more/less resistance to some, not others)?
  - Listen for concerns about AZ vs mRNA vaccines; vaccine mixing; concerns about side effects, etc. and probe accordingly.

**4.** Let’s switch tracks a bit – how do you find your approach to potentially challenging conversations about vaccines, like COVID-19 vaccines, to other requests that patients might come in to see you for, such as tests/procedures or an Rx, where you feel there may be low-value (little benefit) to the patient. Do you feel you have the necessary tools or supports (like strategies, resources, materials) to have effective conversations with patients about those situations? Can you think of a recent example?

*Probe/listen for if relevant based on participant response to the open-ended question above.*

- How do you balance the research-based evidence with your own clinical experience?
- Does/would this hold for all patients, or to what extent might tailoring be required for different patients?
- What about for patients who you might not have a relationship with (and are thus less familiar with their individual/social history) – do you vary that approach or does it influence your clinical recommendation?
- What about for patients with potentially more co-morbidities?
- Are there other factors that might affect your ability to have these conversations? [If participant doesn’t raise anything, consider following up with a probe to ask if time during the appointment or managing a walk-in scenario has any impact?]

## Trust

[NOTE: at this stage in the pandemic – in January to March 2022, patients wanting vaccines were already accessing/had accessed them – but providers were tasked with trying to boost immunization rates for those who had not yet accepted COVID vaccines – so the next few questions are written in a way to explore aspects known in the trust literature to have an influence on acceptance of a recommendation (expressing shared values – that clinical recommendations are motivated by what is believed to be their expert judgement about what is best for the patient in front of them, while still being respectful of patient views).]

**5.** As a clinician, patient care is a priority. However, have you ever had patients directly challenge you or make accusations about you (or the broader health system) about questionable motives when you provided your clinical recommendation – either about a COVID-19 vaccine or a low-value test, procedure or Rx?

[ If participant struggles with what is meant by potentially “questionable motives” a patient might raise, offer some examples - (e.g. financial incentives – either the “money” the provider makes in giving vaccines or choosing one therapy over another, government over-reach (mandates/vaccine-‘passports’, providers being told by government what to do), other conspiracies, issues, etc.])

NOTE: these following questions only were asked if they were needed to build on the general opening question, or if based on the first probe (a) it was relevant to ask probing questions b or c.

**a.** How easy has it been for you to convey to your patients that your clinical recommendations are motivated by what you think is best for their health, while at the same time, communicate to them that you think vaccination is the best recommendation while still leaving room to address their concerns?

**For Vaccines -** How far do you go in challenging conspiracies/challenges (e.g. telling them that we already have vaccine ‘passports’ for childrens’ vaccines for things like daycares and schools)?

**For situations about non-recommended test/procedure/treatment** – have you tried to balance your discussion of risks to the patient relevant to the benefits the knowledge might provide? Or have you ever tried to put those discussions into the context of costs to the health system or delays for other patients who might need to access those diagnostic tests more (e.g. in case of CTs, MRIs, etc).

**b**. Have you ever tried approaches like this? [If yes, what did they think worked well in that conversation and what did they think did not work well?]

**c.** If you’ve ever tried to provide ‘corrective’ information to a patient, how have they received it? Has anyone ever ‘doubled-down’ on their resistance?

**6.** In the case of vaccinations (COVID-19 or otherwise) and the dialogue it can provoke between you and a patient – and recognizing that all situations/patients are different: what is your typical spiel/routine for managing those discussions?

**[***Only probe with more directed questions below depending on how participants answer the general question***]**

**a.** How do you handle giving information (and if necessary, direct: Do you ask if the patient wants information (i.e. are open to listening), or do you jump in with information?

**b.** How do you assess patient preferences? (and if necessary, direct: Do you give some time for the patient to express their preferences of what they want out of the situation – how do you invite that conversation? In your experience do you find some patients want to state their preferences and others not?)

**7.** [*building on what participants have described already in the interview – so feeding back to them what you have heard before asking]* When having conversation about vaccination generally or COVID-19 vaccines, can you identify any additional barriers and facilitators that made those conversations less or more manageable

*Probe for* *examples like - time, personality types (e.g. patients who are info seekers or less active in seeking information), patient aids, ‘leverage-able’ thematic examples/anecdotes/analogies)?*

8. Is there anything else that you would like to add that you don’t feel we have touched on?

[Close with thanking participant, explaining next steps, etc.]
